# Light Spectral Composition Modifies Polyamine Metabolism in Young Wheat Plants

**DOI:** 10.3390/ijms23158394

**Published:** 2022-07-29

**Authors:** Magda Pál, Kamirán Áron Hamow, Altafur Rahman, Imre Majláth, Judit Tajti, Orsolya Kinga Gondor, Mohamed Ahres, Fatemeh Gholizadeh, Gabriella Szalai, Tibor Janda

**Affiliations:** 1 Eötvös Loránd Research Network, Centre for Agricultural Research, 2462 Martonvásár, Hungary; hamow.kamiran@atk.hu (K.Á.H.); altafk013@gmail.com (A.R.); majlath.imre@atk.hu (I.M.); tajtijuci@gmail.com (J.T.); gondor.kinga@atk.hu (O.K.G.); mohamed.ahres@atk.hu (M.A.); fatima.gholizadeh64@gmail.com (F.G.); szalai.gabriella@atk.hu (G.S.); janda.tibor@atk.hu (T.J.); 2 Doctoral School of Horticultural Sciences, Hungarian University of Agriculture and Life Sciences, 2100 Gödöllő, Hungary; 3 Department of Plant Production and Genetics, Faculty of Agriculture, University of Kurdistan, Sanandaj 66177-15175, Iran

**Keywords:** blue light, light quality, putrescine, red light, spermidine, spermine, wheat

## Abstract

Although light-emitting diode (LED) technology has extended the research on targeted photomorphogenic, physiological, and biochemical responses in plants, there is not enough direct information about how light affects polyamine metabolism. In this study, the effect of three spectral compositions (referred to by their most typical characteristic: blue, red, and the combination of blue and red [pink] lights) on polyamine metabolism was compared to those obtained under white light conditions at the same light intensity. Although light quality induced pronounced differences in plant morphology, pigment contents, and the expression of polyamine metabolism-related genes, endogenous polyamine levels did not differ substantially. When exogenous polyamines were applied, their roborative effect were detected under all light conditions, but these beneficial changes were correlated with an increase in polyamine content and polyamine metabolism-related gene expression only under blue light. The effect of the polyamines on leaf gene expression under red light was the opposite, with a decreasing tendency. Results suggest that light quality may optimize plant growth through the adjustment of polyamine metabolism at the gene expression level. Polyamine treatments induced different strategies in fine-tuning of polyamine metabolism, which were induced for optimal plant growth and development under different spectral compositions.

## 1. Introduction

Light is the major energy source for plants and one of the most important environmental factors influencing plant morphology, physiology, and development [1]. Several studies have already been published on the effects of light intensity not only on plant growth and development but also on metabolite accumulation [2,3,4,5,6]. However, besides light intensity, the spectral composition also influences plant growth, development, and stress tolerance. In addition, the specific wavelengths of light have different effects on plants [7,8,9,10,11,12,13,14,15,16,17,18,19]. The synergistic effects of an optimum ratio of red and blue light on the control of plant growth and development have also been demonstrated [20,21,22,23,24]. Besides primary metabolism, light spectra also influence secondary metabolism, including the accumulation of anthocyanins, carotenoids, and flavonols [25,26,27,28]. However, other groups of protective compounds are also influenced, as has been reported in the case of ascorbic acid or proline [29,30,31]. Changes in the spectral composition also modified both the amount and the ratio of free amino acids and the total glutathione pool in wheat plants [32,33,34]. Most of the results are available on the effect of changes in the red/far-red light ratio, but blue light-induced responses are less well known.

Polyamines (PAs) are generally considered plant growth regulators, and they are involved in a range of growth and developmental processes from embryogenic development, stem elongation, root growth, and flower induction to fruit ripening, in addition to having a role in several cell processes, such as gene expression, translation, cell proliferation, membrane stabilization, and even programmed cell death [35,36]. There is also several evidence on the close relationship between PA metabolism and photosynthesis. High level of PAs in chloroplasts, especially in light-harvesting and PS II complexes and the high activity of the transglutaminase enzyme, which is responsible for the covalent binding of PAs to pigment proteins, suggests the involvement of PAs in plant growth via photosynthesis [37,38,39,40]. It has been also proven that light itself controls PA metabolism, both on the synthesis side [33,41,42,43] and on the catabolism side [43,44,45]. Nevertheless, PAs are able to support photosynthesis at several levels: modulating chlorophyll destruction and/or biosynthesis, stabilizing the conformation of photosynthetic complexes, and increasing chemiosmotic ATP synthesis [46]. Earlier, it was accepted that interactions of PAs with macromolecules constituted the main mechanism by which these compounds play their role. Now, the picture is becoming clearer, and it is evident that PAs also interact with phytohormones and other small protective compounds, and thus they have a distinctive role in a complex metabolite and signaling pathway.

Increased nitrate metabolism under blue light conditions has been found to be parallel with higher chlorophyll content and net photosynthetic rate compared to white light conditions [47]. The effects of different light spectra on protein profile and PA content have been found in *Cedrela fissilis*, which in turn affects in vitro shoot development [48]. Similarly, LED lamp treatment with white plus low blue and deep red resulted in greater elongation and higher free PA contents than a fluorescent lamp in *Cariniana legalis* [49]. In addition, when endogenous PA contents were compared between lettuce cotyledon explants cultured under different light qualities, it was found that during shoot primordium production, white or red light conditions resulted in a higher accumulation of free or perchloric acid (PCA)-soluble conjugated PAs and a lower proportion of PCA-insoluble conjugated PAs than under blue light. The ratio of putrescine (PUT) to spermidine (SPD) is lower under white or red light and higher under blue light [50]. Red light also promoted the formation of embryogenic calli in cotton at the induction phase, where the highest total PA contents were detected, while blue light inhibited embryogenic callus formation and resulted in lower PA contents than red light [51].

The relationship between PAs and plant growth and development is evident [52]. In addition, light-related regulation of the PA metabolism in plants was partly reviewed recently [53]. However, there is still a lack of detailed information on how light quality influences PA metabolism. Few studies are available on the comparison of changes in dedicated components of PA metabolism under different spectral conditions. Nevertheless, it is difficult to compare these studies, as different plant species were studied under different light intensities and spectral compositions. The effect of light sources high in blue, blue and red, or far-red irradiance on PA content can be dependent on the light intensity [54]. In addition, in most of the studies only a short period or a pulse of different light spectral conditions were applied on etiolated plants or after dark adaptation [44,55,56], and the results were obtained mainly from *Arabidopsis* plants (as reviewed in [53]).

We have demonstrated that, different light quantities (either the differences in the hours of daily illumination or the light intensities) induced different changes in polyamine metabolism. Briefly, light distinctly induced the PUT level and reduced the 1,3-diamino propane (DAP) content in the leaves, the latter result suggesting the inhibition of the terminal catabolism of higher PAs under higher light conditions. In addition, exogenous PA treatments influenced the PA metabolism differently under altered light intensities [57]. However, despite the above-described information about the relationship between PA metabolism and light, there is still little direct information available about light-influenced PA metabolism. Until now, there has been no literature about the relationship between light and PA metabolism under not only different spectral conditions but together with the application of exogenous PAs. In the present study. the effects of three spectra were studied using wheat plants and compared to the control—white light at the same light intensity—to answer the following questions: (1) How do the light conditions, referred to by their typical characteristics (blue, red + far-red, and the combination of blue and red + far-red, called pink lights) influence PA metabolism?; (2) How do these light conditions influence the effects of exogenous PAs compared to each other?; and (3) How do PA treatments modify the effect of different spectral conditions at the metabolite and gene expression levels?

## 2. Results

Four light conditions were used in the present experiment. The light source for white light (W) was a continuous wide-spectrum LED. In the case of the blue regime (B), blue light was dominant (82.55%) and its ratio was approximately 5 times greater than the red light. In the case of the red regime (R), the red light was dominant (66.28% red and 6.59% far-red), as its ratio was approximately 5 times greater than that of the blue light, while in the pink (P) regimen, the ratio of blue and red was approximately 1 (see in the Materials and Methods section for the main characteristics of light regimes). Results were compared, with special regard to highlighting the differences between the B-, R-, and P light-induced changes in comparison to the white light-related ones. PA treatments, namely PUT, SPD, and spermine (SPM) were applied at 0.3 mM concentration only under B, R, and P light regimes. In a previous study, the effects of these PA compounds were investigated under white light conditions at different light intensities [57]. In the present study PUT, SPD, and SPM treatments were applied only under B, R, and P regimes.

### 2.1. The Effect of Different Spectral Compositions in Combination with Polyamine Treatments on Physiological Parameters, Proline Content, and Plant Hormone Composition

#### 2.1.1. Changes in Chlorophyll-*a* Fluorescence Induction Parameters and Pigment Contents

**Figure 1:**
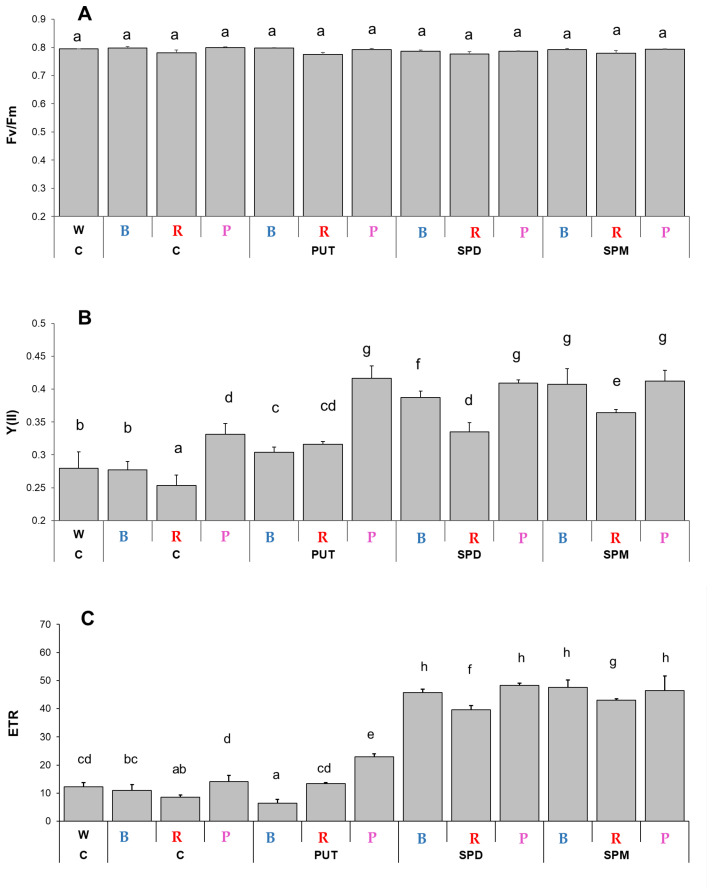
Cholorphyll-*a* fluorescence induction parameters. (**A**): Maximum quantum yield of PSII (F_v_/F_m_), (**B**): actual quantum yield of PS II [Y(II)], and (**C**): the electron transport rate (ETR) determined at the steady state level of photosynthesis on the third fully expanded leaves of plants grown under different light regimes [(white light (W), blue light (B), red + far-red: R and the combination of blue and red + far-red, called pink (P)] treated with or without polyamines (control: C, putrescine: PUT, spermidine: SPD and spermine: SPM). Values are means ± SD (*n* = 6). Different letters indicate statistically significant differences at *p* < 0.05 level, using Duncan’s post hoc test.

Chlorophyll-*a* fluorescence quenching analyses revealed that although the light quality did not influence the maximum quantum yield of PS II (Fv/Fm parameter, Figure 1A), it affected the photosynthetic activity of PS II, as significant differences were detected in the actual quantum yield [Y(II)] and the electron transport rate (ETR) (Figure 1B,C). Blue light illumination resulted in similar Y(II) value, as it was detected under W light, while the lowest value of Y(II) was found under R light and the highest under P light (Figure 1B). The positive effect of 0.3 mM PA treatments was detected under all of the B, R, and P light conditions, and it was the most pronounced in the case of SPM under B and R lights, with 147% and 143% increments, respectively, compared to the controls. Similar changes and differences were found in the case of the ETR parameter (Figure 1C). The lowest ETR value was detected under R conditions. SPM treatment induced the highest increase in the ETR parameter under both B and R light conditions compared to the control ones, as the ETR value reached the highest level at P illumination during control conditions (Figure 1C).

The results on fluorescence induction parameters showed similar changes with the plant pigment contents (Table 1). Light quality differently affected all the presented pigment levels. B light increased all of them except for *trans*-neoxanthin, while R light caused similar or lower concentrations as W itself. In almost all cases, under the P regime, the diminishing effect of red light was partly compensated by the presence of blue light. The roborative effect of exogenous PAs has been also recognized here in the results of plant pigment content, especially in the cases of *trans*-lutein, chlorophyll *a*, and chlorophyll *b* after SPM treatment.

#### 2.1.2. Differences in Biomass Parameters

Wheat plants grown under W light conditions exhibited the highest elongation in both the shoots and roots (Table 2). Blue light treatment inhibited elongation, but due to the resulting compact morphology, the shoot and root weights did not differ pronouncedly compared to the control plants grown under W light. Compared to the W regime, the R light without PA treatment had minor effects on elongation parameters, whereas under P light, the effect of the blue component in the spectral composition was still dominant in the reduction of elongation, which could not be mitigated by increase of the red light ratio. The effect of blue light on shoot and root weights was also dominant over the effect of red under the P regime, as similar biomass values were measured in the case of B light alone. Interestingly, PA treatments in several cases induced significant changes in the biomass parameters, but these differences were slight only. In addition, shoots and roots responded partly differently to the various treatments. In general, biomass values increased in the shoots, especially after SPM treatment, while in the roots higher PAs (SPD and SPM) induced no change or even decrease in these parameters.

#### 2.1.3. Changes in Proline Content

**Figure 2:**
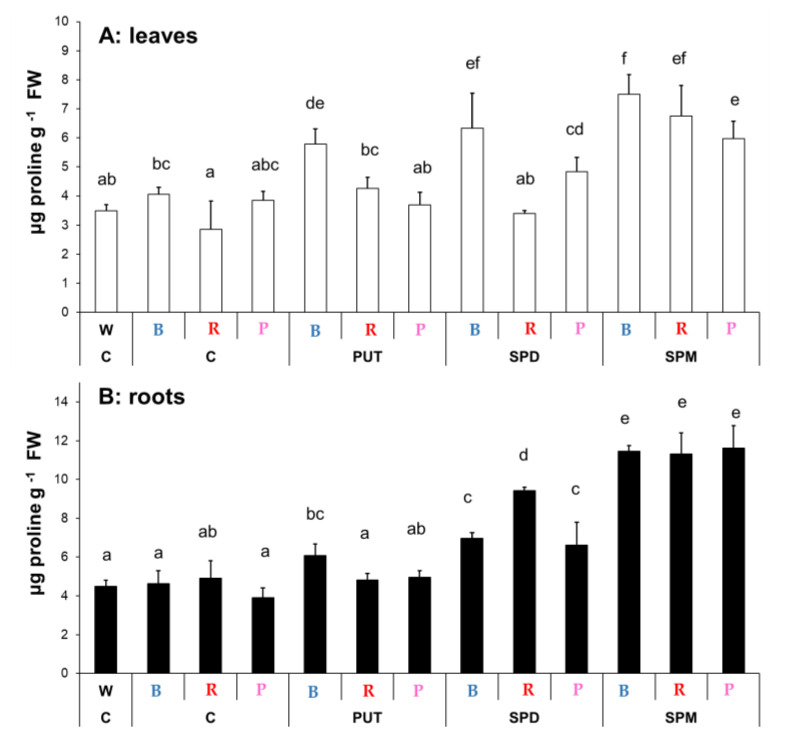
Changes in proline contents in the leaves (**A**) and roots (**B**) of plants grown under different light regimes (white light: W, blue light: B, red + far-red: R and the combination of blue and red + far-red, pink: P) treated with or without polyamines (control: C, putrescine: PUT, spermidine: SPD and spermine: SPM). Values are means ± SD (*n* = 3). Different letters indicate statistically significant differences at *p* < 0.05 level, using Duncan’s post hoc test.

Light quality (B, R, or P) had no significant effect on the proline content in the leaves or roots compared to the W light condition (Figure 2). However, the proline content of the leaves was lower in plants grown under R light condition, in contrast to those, which were treated with blue light. Under blue light, all the PA treatments increased the proline level in the leaves, but under R or P regimens, significant increases were only detected after SPM application. In the roots, under B light, PUT, SPD and SPM increased the proline level, while under R and P lights, only SPD and SPM treatments could increase it (Figure 2A,B).

#### 2.1.4. Changes in Salicylic Acid and Abscisic Acid Contents

Previous studies demonstrated, that PA treatments influence the synthesis of certain plant hormones. In addition, light may have also influence on their levels. In order to further characterize the physiological status of wheat plants, the levels of two plant hormones, salicylic acid (SA) and abscisic acid (ABA), were determined.

**Figure 3:**
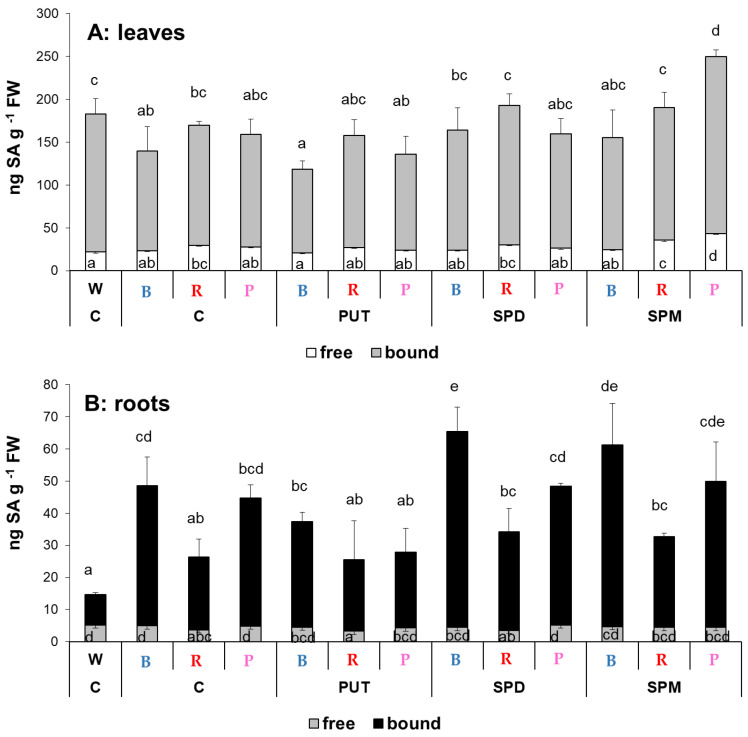
Salicylic acid (SA) contents in the leaves (**A**) and roots (**B**) of plants grown under different light regimes (white light: W, blue light: B, red + far-red: R and the combination of blue and red + far-red, pink: P) treated with or without polyamines (control: C, putrescine: PUT, spermidine: SPD and spermine: SPM). Values are means ± SD (*n* = 3). Different letters indicate statistically significant differences at *p* < 0.05 level, using Duncan’s post hoc test.

Although the light composition has no pronounced effect on the free or bound SA content in the leaves, interestingly in the roots significant differences were detected in the bound fraction, resulting in increased total SA content either under B, R, or P light compared to the W light condition (Figure 3A). Statistically significant effects of exogenous PAs on total leaf SA level could not be recognized, except for when the SPM-treated plants were compared to the control under the P regime. The root SA amount increased after SPD and SPM treatments under the B regime, and no significant changes were detected under R or P light conditions (Figure 3B).

**Figure 4:**
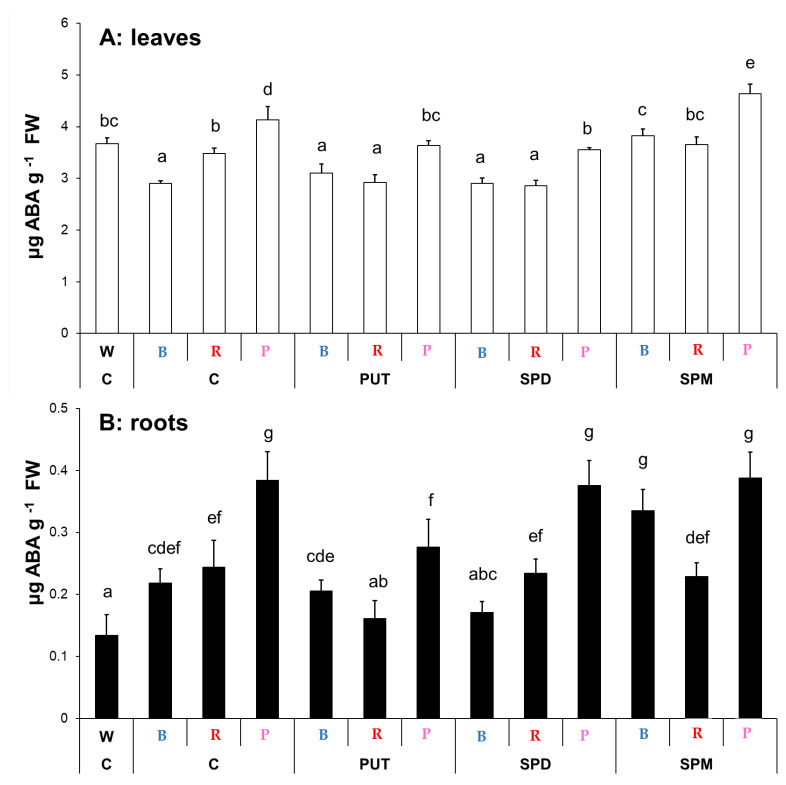
Abscisic acid (ABA) contents in the leaves (**A**) and roots (**B**) of plants grown under different light regimes (white light: W, blue light: B, red + far-red: R and the combination of blue and red + far-red, pink: P) treated with or without polyamines (control: C, putrescine: PUT, spermidine: SPD and spermine: SPM). Values are means ± SD (*n* = 3). Different letters indicate statistically significant differences at *p* < 0.05 level, using Duncan’s post hoc test.

The effect of light composition affected the leaf ABA content. The lowest level was found with the B light treatment, and the highest level was detected under the P regime. The W and R regimes had similar ABA levels (Figure 4A). PA treatments also modified the leaf ABA content depending on the light conditions, indicating an interaction between light and PA signaling. Under B light, only SPM could increase its level, under R light PUT and SPD treatments decreased it, while under the P regime PUT and SPD also decreased, but SPM increased it (Figure 4A). In the roots, ABA content was also influenced by the light conditions compared to the W light. All of the special light regimes increased it, with the highest accumulation under P light (Figure 4B). Interestingly, only SPM treatment increased its level under B light, while under R and P conditions a decrease in it was detected after PUT treatment. Changes in the levels of ABA showed similar patterns under R and P regimes after PA treatments in both the leaves and roots, respectively (Figure 4).

### 2.2. Effects of Different Spectral Conditions in Combination with Polyamine Treatments on Polyamine Metabolism

#### 2.2.1. Changes in Polyamine Contents

**Figure 5:**
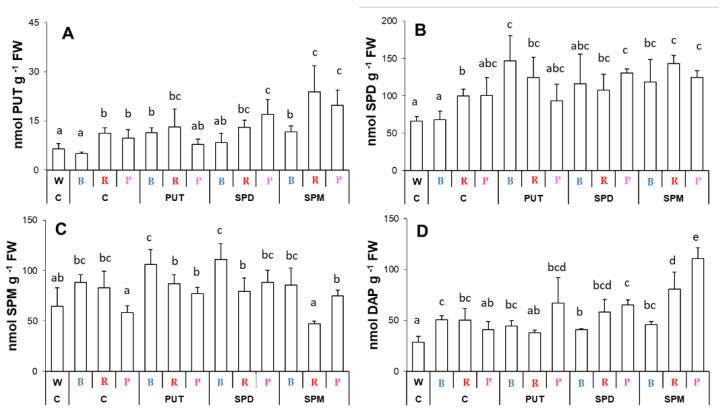
Polyamine contents ((**A**): putrescine: PUT; (**B**): spermidine: SPD; (**C**): spermine: SPM; (**D**): 1,3-diaminopropane: DAP) in the leaves of plants grown under different light regimes (white light: W, blue light: B, red + far-red: R and the combination of blue and red + far-red, pink: P) treated with or without polyamines (control: C, putrescine: PUT, spermidine: SPD and spermine: SPM). Values are means ± SD (*n* = 3). Different letters indicate statistically significant differences at *p* < 0.05 level, using Duncan’s post hoc test.

Compared to the white light conditions, B light treatment did not influence the PUT, SPD, or SPM contents, but increased DAP level in the leaves (Figure 5A–D). Though besides DAP content, R light also significantly increased the amount of PUT and SPD, the P light condition did not modify significantly the PA levels. The exogenous PAs induced changes in the endogenous PA levels with a different efficiency under B, R, or P light treatment. The PUT level slightly increased after all of the applied PA treatments under B light, but only after SPM treatment under R light condition compared to their controls. P light condition combined with SPD or SPM treatment also increased PUT level (Figure 5A). Pronounced increment of SPD amount was also found after PA treatments under B condition, while only slight increases were observed under R or P conditions (Figure 5B). Leaf SPM content did not change significantly after PA treatments under B light, but increased after PA treatment under P and decreased by SPM treatment under R conditions (Figure 5C). Interestingly, the amount of DAP showed an increasing tendency after SPM treatment only under R and P conditions (Figure 5D), suggesting that the catabolism induced by the excess of higher PAs in the leaves is a result of the presence of a higher proportion of red light.

**Figure 6:**
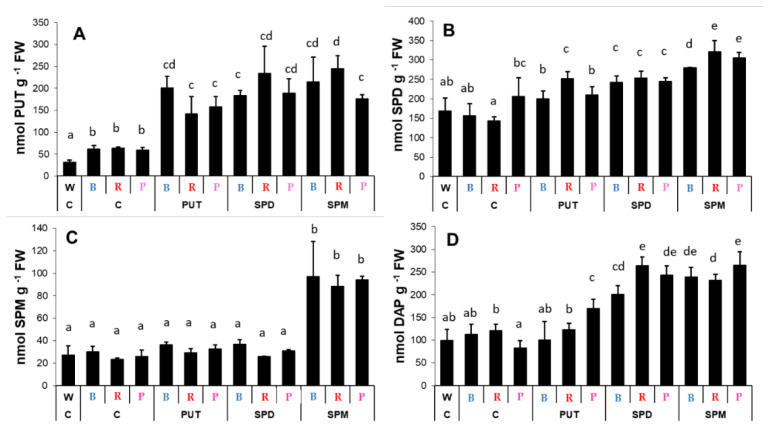
Polyamine contents ((**A**): putrescine: PUT; (**B**): spermidine: SPD; (**C**): spermine: SPM; (**D**): 1,3-diaminopropane: DAP) in the roots of plants grown under different light regimes (white light: W, blue light: B, red + far-red: R and the combination of blue and red + far-red, pink: P) treated with or without polyamines (control: C, putrescine: PUT, spermidine: SPD and spermine: SPM). Values are means ± SD (*n* = 3). Different letters indicate statistically significant differences at *p* < 0.05 level, using Duncan’s post hoc test.

Light treatment itself did not influence the endogenous PA levels in the roots, while exogenous PAs had pronounced effects on them (Figure 6). Characteristic changes were induced in the PUT, SPD, SPM, and DAP contents after PA applications (Figure 6A–D), especially after SPM treatment. However, these tendencies were very similar to each other under all light conditions.

#### 2.2.2. Differences in the Expression Levels of Genes Involved

**Figure 7:**
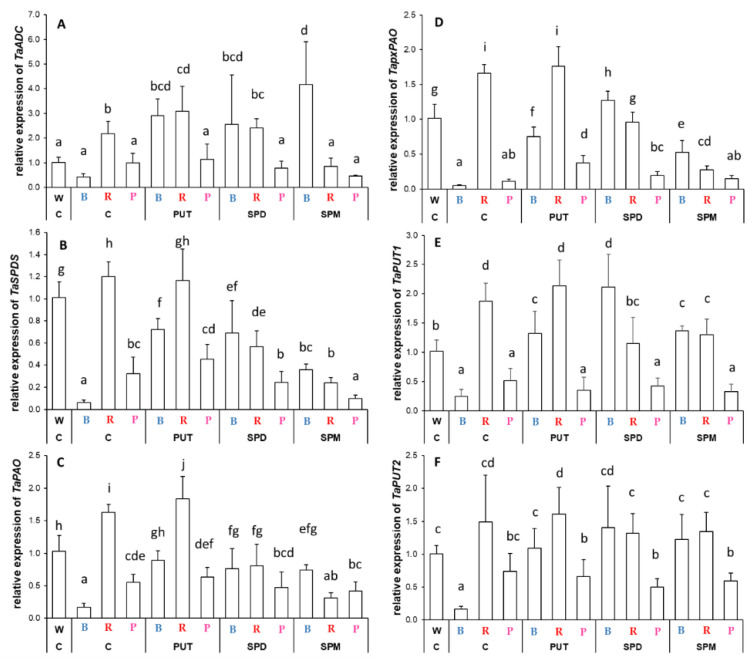
Gene expression patterns of arginine decarboxylase ((**A**): *ADC*), spermidine synthase ((**B**): *SPDS*), peroxisomal polyamine oxidase ((**C**): *pxPAO*),polyamine oxidase ((**D**): *PAO*) and polyamine uptake transporter genes ((**E**): *PUT1* and (**F**): *PUT2*) in the leaves of plants grown under different light regimes (white light: W, blue light: B, red + far-red: R and the combination of blue and red + far-red, pink: P) treated with or without polyamines (control: C, putrescine: PUT, spermidine: SPD and spermine: SPM). Values are means ± SD. All reactions for gene expression analyses were performed in triplicate using 3 biological and 3 technical repetitions. Different letters indicate statistically significant differences at *p* < 0.05 level, using Duncan’s post hoc test.

Different light compositions caused statistically significant and characteristic patterns in the expression pattern of PA metabolism-related genes in the leaves (Figure 7). Without PA treatments, only R light could influence arginine decarboxylase (*ADC*) expression, increasing it (Figure 7A). Under R condition, the PUT content slightly increased *ADC* expression, too. Under B light, all the PA treatments induced its transcription to a similar level as was found in the control plants under R conditions, whereas under R conditions, the SPM treatment decreased its transcription. Interestingly, the PA treatments had no effect under P light (Figure 7A). B light or P light decreased spermidine synthase (*SPDS*) expression—with higher decrease in the case of the B regime—compared to that observed in plants grown under W light conditions, while R slightly increased it (Figure 7B). According to these, the influence of PA treatments on *SPDS* expression was also different under the three light regimes. Under B light, all the PAs increased *SPDS* expression, but this activation was not enough to reach the level of expression found under W light conditions. Under R light, SPD and SPM treatments decreased it, while under P light, SPM application also decreased its expression with a similar pattern (Figure 7B). 

The transcript level of *PAO*, responsible for the terminal catabolism of SPD and SPM, also showed basal light dependence in the leaves (Figure 7C). As B light decreased, R light increased it, while P light also decreased it compared to those determined in the plants grown under W light. Its expression level increased by all PA treatments under the B regime, resulting in similar values as in plants grown under W light, but it was decreased by SPD and SPM treatments under R regime. Neither of the PA treatments could change the expression level significantly under P light conditions.

A partly similar expression pattern was observed for peroxisomal *PAO* (*pxPAO*) as was described for *ADC*, *SPDS*, and *PAO* (Figure 7D). B light alone decreased the transcription of *pxPAO*, but after PA treatments it was activated, and almost resulted in similar expression to that found in plants under W light. Under the R regime, the initially higher expression was inhibited by SPD and SPM application, while P light alone decreased it, and only PUT treatment could increase its expression (Figure 7D).

The effect of B light was also characteristic on the polyamine uptake transporter genes (*PUT1* and *PUT2*) (Figure 7E,F). Under B and P light conditions, the transcript level of *PUT1* and *PUT2* was lower than under W or R conditions, but PA treatments could only induce their expression under B light treatment.

**Figure 8:**
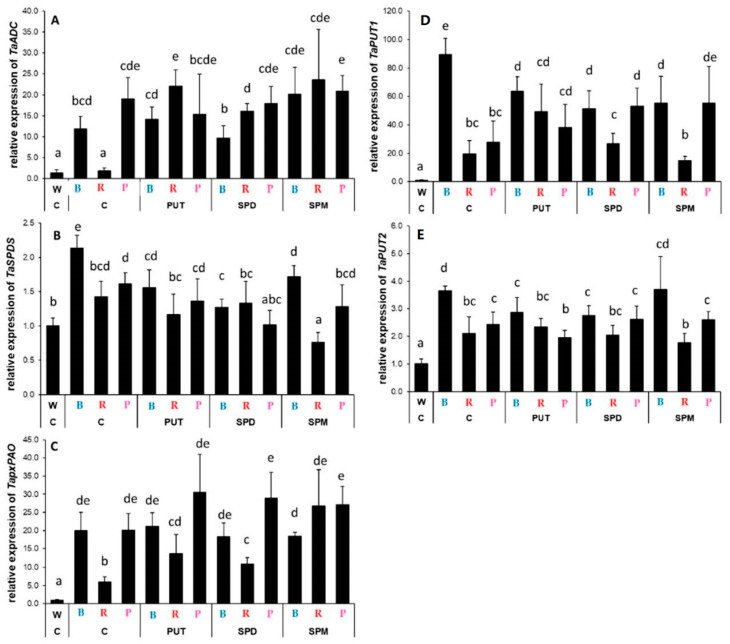
Gene expression patterns of arginine decarboxylase ((**A**): *ADC*), spermidine synthase ((**B**): *SPDS*), peroxisomal polyamine oxidase ((**C**): *pxPAO*) and polyamine uptake transporter genes ((**D**): *PUT1* and (**E**): *PUT2*) in the leaves of plants grown under different light regimes (white light: W, blue light: B, red + far-red: R and the combination of blue and red + far-red, pink: P) treated with or without polyamines (control: C, putrescine: PUT, spermidine: SPD and spermine: SPM). Values are means ± SD. All reactions for gene expression analyses were performed in triplicate using 3 biological and 3 technical repetitions. Different letters indicate statistically significant differences at *p* < 0.05 level, using Duncan’s post hoc test.

Interestingly, a completely opposite effect of blue light was observed on PA metabolism-related gene expression in the roots compared to that found in the leaves. Without any PA treatment among light treatments, the B light induced the expression of *ADC*, *SPDS*, *pxPAO*, *PUT1*, and *PUT2* genes compared to that detected under W or R conditions, while the effects of the P light treatment were rather similar in cases of *ADC* and *pxPAO* to that of B light treatment, but in cases of *SPDS*, *PUT1*, and *PUT2* the transcript levels were similar to those measured under R light conditions (Figure 8A–E). Even though the effect of blue light on gene expression levels could be detected in the leaves under P light treatment, it was only true for *ACD* and *pxPAO* in the roots. Nevertheless, the PA treatments had different effects depending on the investigated gene and the applied spectral composition. Exogenous PA application could induce the *ADC* gene expression under R conditions (Figure 5A) and the transcription of *SPDS* slightly, but it was tendentiously decreased by PAs under all the light treatments (Figure 8B). The level of *pxPAO* gene expression increased with the PA treatments under R and P light conditions, but it was more pronounced with R treatment (Figure 8B). The expression of *PAO* was not detectable in the roots.

The transcript levels of *PUT1* were greatly increased by blue light, but it was decreased after PA treatments (Figure 8D). PUT application under R treatment, while all the PA treatments under P condition increased it. Similarly, *PUT2* expression was induced especially by blue light compared to the W light conditions, while PA treatments had no pronounced influence on it under either light condition (Figure 8E).

## 3. Discussion

Besides the light intensity and duration of illumination, i.e., light quantity, specific light qualities also have deep effects on plants. Light spectral composition can influence plant morphology, physiology, development, and stress responses by impacting on processes ranging from photosynthesis to secondary metabolism [58]. As blue and red light are the most effectively utilized wavelengths during photosynthesis, the understanding of their regulatory role in other plant processes is an important aspect.

Generally, it can be said that red light plays an important role in controlling chloroplast functions, stem and petiole growth, and reproductive development, while blue light influences plant growth, leaf expansion, photomorphogenesis, stomatal opening, photosynthesis, and pigment accumulation [22]. Far-red light accelerating plant flowering can also modulate plant height and leaf size, thus regulating plant morphology and photosynthetic capacity, enabling plants to capture more light and in turn indirectly increase growth [59]. Although several studies have focused on evaluating the effects of light composition, some specific processes affected by spectral changes remain largely unknown.

Despite the numerous studies on the role of PAs in plant growth and development and on the protective and beneficial effects of various PA treatments (seed soaking, hydroponically or spraying) in stress responses and tolerance [60,61,62,63,64,65], there is still little direct and detailed information available about how light influences PA metabolism and consequently its effects. Most of the studies on the investigation of the effect of light quantity were performed on *Arabidopsis* mutants, under different light intensities or with only one spectral composition compared to white light conditions for a short period or after dark adaptation. In addition, even fewer studies are available focusing directly on PA metabolism under different spectral conditions, as above [reviewed in 53]. In the present study, the effect of three different spectral compositions were studied in comparison with white light conditions. Besides some photosynthesis related and biomass parameters, the contents of proline and plant hormones (SA and ABA)—which usually show a close relationship with PA levels—were also determined, but the present study focused mainly on PA metabolism at metabolite and gene expression levels. In our previous study, the effects of PA treatment under white light conditions was investigated under different light intensities, where it was demonstrated that in the leaves, light distinctly induced the PUT level and reduced the DAP content, which suggests that higher light conditions inhibit the terminal catabolism of higher PAs. In addition, exogenous PA treatments influenced the PA metabolism differently under different light intensities [57]. In the present study the effects of PUT, SPD, and SPM treatments were only investigated under B, R, and P regimes.

### 3.1. Light Quality Modifies Certain Photosynthesis- and Biomass-Related Parameters and Plant Hormone Levels

Under the present conditions, B light had no significant effect on the investigated fluorescence induction parameters, Y(II) and ETR, indicating the presence of a control mechanism that regulates the electron transport process. However, shorter and more compact plant morphology was observed (as indicated by the biomass parameters), as well as higher pigment concentrations, particularly chlorophyll a and b, *trans*-violaxanthin, and β-carotene contents. In contrast, the dominant ratio of red decreased the PSII actual quantum yield and the electron transport rate, although the pigment content pattern was similar, and the biomass parameters were also almost the same as found in the plants grown under white light conditions. At the same time, P light treatment increased the photosynthesis-related Y(II) and ETR parameters, the chlorophyll a and b contents, and the β-carotene levels, although decreased shoot and root length values were detected compared to plants grown under white light conditions. These findings are in accordance with the results of other studies on barley [66], sweet pepper [22], and wheat [32]. However, responses of different plant species can be partly different. Various combinations of red and blue light have been investigated [24].

The spectral composition did not influence pronouncedly the proline content in the leaves and roots or the SA levels in the leaves compared to the W light conditions. However, the root SA content was increased under B and P light treatments compared to the W or R conditions. In addition, although the ABA concentration slightly decreased in the leaves under B light, it increased remarkably under P light treatment in both the leaves and roots. In *Drosera peltata* and *Dionaea muscipula* in vitro cultures, blue–red LED light (spectral composition ratio 6:1) treatment resulted in high accumulation of SA in both plant species, but did not influence the proline contents compared to the fluorescent light conditions [67]. However, in wheat flag leaves blue light induced and far-red light decreased the proline content [32], but an opposite tendency was observed in the leaves of 10-day-old wheat plants [33]. In watermelon, comparison of LEDs emitting narrow-band red, narrow-band blue, or combination of red and blue light (88:12) revealed that the combined treatment induced the highest ABA content, but the red light condition resulted in the highest SA accumulation in both the scion and the rootstock [68]. Different combinations of blue and red light induced the growth of *Salvia miltiorrhiza*, which was related to enhanced accumulation of phenolic acids and activation of *PAL1* gene expression, the gene encoding phenylalanine ammonia-lyase enzyme, which also has a key role in SA synthesis [69]. Similarly to our results, the increasing ratio of blue light also decreased the ABA content in rose leaves under light conditions [70], while far-red supplementation increased ABA levels in barley [71]. However, due to the widespread use of LED light sources, knowledge of the effects of spectral composition on phytohormone metabolism is an emerging field.

### 3.2. Light Quality May Influence the Expression of Genes Related to Polyamine Metabolism without Affecting the Polyamine Levels

Despite the pronounced differences observed visually and detected in the biomass parameters, plant pigment contents, and fluorescence induction parameters, B, R, or P light treatments caused only slight changes in the endogenous PA contents in the leaves and did not influence them in the roots. The lowest leaf PUT level was detected under B light treatment and was higher under R or P treatments than under W light. The content of SPD was similar under B and W treatments, while it was higher under R and P conditions. The SPM levels were higher under B and R conditions than under W or P light conditions, while the DAP amount increased especially during B and P treatments. These results suggest that due to dynamic PA metabolism, the balance of the synthesis, catabolism, or back-conversion resulted in such similar PA levels under light conditions that are optimal for the adequate plant growth conditions.

Investigation on the effect of different light sources on in vitro shoot development in *Cariniana legalis* revealed that the combination of white, low-blue, and deep-red without far-red spectra resulted in higher levels of PUT, SPD, and total free PAs compared to the fluorescent light conditions [50]. In *Cedrela fissilis* Vell., LED light with white plus medium blue and deep red spectral composition induced only a slight increase in PUT content, but did not influence the SPD or SPM content compared to the fluorescent light treatment [49]. Also in wheat plants, the level of ornithine, one of the precursor compounds of PUT synthesis, was hardly influenced by the spectral composition, while the amount of arginine, another precursor of PUT, was highest under far-red light conditions [33]. However, the level of arginine was not in accordance with the gene expression level of *ADC*, as it was decreased by both blue and far-red light treatment compared to that measured under white light conditions [33]. In *Arabidopsis*, the comparative gene expression analysis of RNA isolated from the plants grown under LED light supplemented with blue, red, or the combination of blue and red revealed that PA synthesis was not affected [72]. In contrast, under the present conditions, the pattern of the expression level of the genes involved in the PA metabolism showed pronounced differences depending on the spectral composition. In addition, the leaves and roots responded differently. In the leaves, the most characteristic effect was observed for the B treatment, as the transcript level of all the investigated genes was the lowest there compared to the other light conditions. Growth under R light resulted in similar gene expression levels as found under white light. Only slight increases were detected, especially in the cases of *ADC*, *PAO*, and *PUT1*. Under P light conditions, the additive effect of blue and red light treatments could be observed, as similar or a slightly lower levels of gene expression were detected compared to those found under W light treatment, so the decreasing effect of blue light on gene expression was partly alleviated by the higher ratio of red in the composition. Interestingly, in the roots B, R, or P light treatments induced the expression of almost all the investigated genes compared to the white light condition. However, here the effect of B light was positive, and the R light induced only similar or slightly higher expression levels as white light. Under the combined P light conditions, the dominant, gene expression-inducing effect of the blue light component was observed for *ADC* and *pxPAO*.

According to these results, it seems that plants try to maintain the same optimal concentration and ratio of PA under different spectral compositions. In addition, the effects of light quantity or time of illumination or the lack of light may be more dominant on the actual PA pool than the light quality [32,33,43,54,57]. However, it should also be taken into consideration that red and far-red light supplementation can result in similar changes as growth under white light conditions, especially under low light intensity [33,50].

### 3.3. PA Treatments Have Roborative Effects under Different Spectral Compositions and Their Effects on PA Metabolism Depend on the Light Quality

According to the present results, the effects of different combination of light spectra on endogenous PA metabolism are still not clear. In addition, the level of PA can alter during development or due to environmental factors leading to different morphological responses [73]. Thus, a well-maintained dynamic balance of PAs is necessary for plant development and stress tolerance. The positive effect of exogenous PAs has been reported in different plant species under control or various stress conditions, and the roborative or protective effect was always related to fine-tuning of PA metabolism [60,61,63,65]. Under the present conditions, the PA treatments increased the electron transport rate around PSII, the photosynthesis-related pigment contents, and in some cases the biomass parameters compared to the control under the same light conditions. Increased chlorophyll-a fluorescence induction parameters and chlorophyll contents showed similar patterns as PUT content in the leaves. Several studies with different plant species provided evidence of the positive effect of PUT on photosynthetic pigments, photosynthetic CO_2_ assimilation, or ATP production [35]. Similarly, the positive effect of PAs has also been demonstrated under different light quantity conditions, where also in wheat, PUT, SPD, or SPM treatment under low or medium light resulted in similar actual quantum yield as was detected under the elevated light condition, where the leaf PUT content also correlated with the Y(II) parameter [57]. The exogenous PA treatments, especially higher PAs (SPD and SPM) also increased the proline content, and especially in the roots, the changes were in relation to the increased total PA level. As biosynthesis of PAs and proline use glutamate as a common precursor, considerable changes in the pool of PAs could cause a shift between the synthesis pathways from PA to proline. Besides acting as an ROS scavenger or osmolite under stress conditions, proline is also a source of nitrogen and carbon, and thus may increase plant growth and development. Positive effects of exogenous PA treatments on biomass parameters have been also reported to be parallel with changes in proline content in the dwarf wheat genotype [63]. Close correlations have been found between SA and PA contents [63], and PA treatments, especially higher PAs, induced the accumulation of SA in wheat and maize leaves and roots [61]. Under the present conditions, a significant increase in leaf SA content was observed under P light in SPM-treated plants, while the root SA level showed an increasing tendency under B and P light after SPD and SPM application. Similarly, a greater effect on roots SA production has been described after PA application in the same wheat genotypes under white light conditions [57]. A positive feedback loop between ABA and PAs has been suggested by several findings [57,64]. Under the present conditions, the ABA level increased under B or P light conditions after SPM treatment in the leaves, and in the roots under B light also after SPM treatment. Under white light, all the PA treatments induced ABA accumulation in the leaves and roots of wheat plants [58]. As ABA and SA synthesis is also regulated by light conditions [74,75], the different spectral compositions may influence the modulating effect of PA treatments on plant hormones differently.

Although the initial leaf PA levels were only slightly different under W, B, R, and P light conditions, the excess of PAs had pronouncedly different effects on PA metabolism under the three spectral compositions. In the leaves, the SPM treatment increased the PUT level under R and P light treatments. However in these treatments, *ADC* expression showed a decreasing tendency as a feedback mechanism. Interestingly, the transcript level of *ADC* under B light increased after all the PA treatments, which was in accordance with the slight increase in PUT levels. Similarly, under B light, excess PA increased leaf SPD levels, and parallel with this, *SPDS* expression also increased. Under R and P light treatment, SPM application increased SPD content, but decreased the *SPDS* transcript level. The SPM level could hardly be influenced, except for SPM-treated plants growth under R conditions, where it decreased. The DAP content also increased after SPM treatment under R and P light conditions. The *PAO* gene, which encodes the apoplastic PAO enzyme, responsible for the terminal catabolism of SPD and SPM, was induced after PA treatments under B light, resulting in similar DAP level as in the control plants, while it was inhibited after SPM treatment under R light where high DAP concentration was found, though no changes in *PAO* expression were observed under P light, maybe as an additive effect of the combination of blue and red light. A similar expression pattern was found for the gene encoding the peroxisomal PAO, responsible for the back-conversion of higher PAs to PUT or SPD. These results suggest that the applied PAs are taken up by the plants, metabolized by terminal catabolism, and back-converted, in addition to being translocated into the leaves. However, as under B light the basal gene expression levels were very low, PA treatments could cause drastic changes in them, both on the synthesis and the catabolism sides. In addition, the same was true for the PA uptake transporter genes (*PUT1* and *PUT2*).

In the roots, due to the hydroponic application, more pronounced changes were detected in the endogenous PA content, especially after SPM treatment. The exogenous SPD and SPM not only elevated their own levels, but due to the back-conversion, the levels of PUT and SPD also increased, leading to terminal catabolism and increased DAP content. However, in contrast to the leaves, the lowest expression levels for most of the investigated genes were found under R treatment. PA treatments induced high expression of *ADC* and *pxPAO* genes only under R light. The transcript level of *SPDS* showed only a slight decreasing tendency after PA treatments under any spectral condition. Expression of *PUT1* decreased under B light after all the PA treatments, increased in PUT-treated and decreased in SPD or SPM treated ones under R light, and increased by the applied PAs under P light conditions.

## 4. Materials and Methods

### 4.1. Plant Materials, Growth Conditions, and Treatments

The wheat seeds (winter wheat (*Triticum aestivum* L.) variety “Mv Béres”) were germinated for 3 days at 26 °C, and thereafter were grown in modified Hoagland solution [76], 15 seedlings per plastic container, and changed every 2 days. Containers were placed in a Conviron PGR-15 plant-growth chamber (Controlled Environments Ltd., Winnipeg, Canada) in a randomized manner, using the following settings: 22/20 °C day/night temperature with 16/8-h light/dark periodicity, with relative humidity of 75%, as described in [76]. Plants from six containers were used for the detailed analyses for every treatment.

Plants were grown under different spectral conditions at the same light intensity (250 µmol m^−2^ s^−1^). Four light regimes were established using modules equipped with a continuous wide-spectrum LED (Philips Lumileds, LXZ2-5790-y) and three narrow bands of LEDs with dominant wavelengths of 448 nm (Philips Lumileds, LXZ1-PR01), 655 nm (Philips Lumileds, LXZ1-PA01), and 750 nm (Edison Edixeon, 2ER101FX00000001). All LED modules were equipped with these LEDs, and each type of LED was independently controlled within the module. The spectral composition used in the experiments—composed of different combinations of LEDs—is described in [32]. The four light conditions are referred to according to their typical characteristic. The light source for white light (W) was a continuous wide-spectrum LED (Philips Lumileds, LXZ25790-y). In the blue regimen (B), the blue light intensity was 5 times that of the red light. In the red + far-red regimen (R), the red light was dominant with elevated far-red component compared with the other light conditions (with 66.28% red and 6.59% far-red light components), and its intensity is approximately 5 times that of the blue one. In the pink (P) regimen, the ratio of blue and red was approximately 1 [32]. The most important characteristics of the light conditions are summarized in Table 3.

The 7-day-old plants (4 leaf development stages) were treated with nutrition solution containing 0.3 mM PUT, SPD, or SPM (based on [50]). After 1-week exposure to different PA treatments, the fully developed leaves and roots were sampled.

### 4.2. Chlorophyll a Fluorescence Induction (FI) Analysis

The FI analysis was carried out using a pulse amplitude modulated fluorometer (PAM) with a blue LED-array illumination unit IMAG-MAX/L (λ = 450 nm) (Imaging-PAM MSeries, Walz, Effeltrich, Germany) on the fully expanded leaves of wheat, which were exposed to dark for 15 min in order to reach the open state of the acceptor side of electron transport chain. Application of a short (800 ms) saturation light (PPFD: 3000 µmol m^−2^ s^−1^) led to the measurement of F_0_ and F_m_ fluorescence and the maximum quantum yield (F_v_/F_m_) of photosystem II (PSII) was calculated. The kinetic (quenching) analysis was carried out to determine actual quantum yield [Y(II)] and electron transport rate (ETR), as described in [77]. The blue actinic light intensity was PPFD: 150 µmol m^−2^ s^−1^. The saturation pulses were set with 30 s frequency in order to record the Y(II) at light-adapted state. The IMAG-MAX/L illumination unit was used throughout the entire quenching analysis (675 s).

### 4.3. Polyamine Analysis

Leaf and root samples were homogenized in 2 mL 0.2 N HClO_4_ and left on ice for half an hour. The homogenates were centrifuged at 4 °C in a centrifuge for 10 min at 10,000× *g*. The supernatant was used for the precolumn derivatization with dansyl chloride, according to [78]. DAP, PUT, SPD, and SPM were analyzed on a reverse phase Kinetex column (C18, 100 × 2.1 mm, 5 μm, Phenomenex, Torrance, CA, USA) by HPLC consisting of a W2690 separation module and a W474 scanning fluorescence detector with excitation at 340 nm and emission at 515 nm (Waters, Milford, MA, USA).

### 4.4. Extraction of Plant Hormones and Analytical Procedure

The extraction procedure and both the separation and detection for ABA with tandem mass spectrometry (UPLC-MS/MS) were carried out according to [57]. Briefly, leaf or root samples were homogenized in liquid N_2_ and extracted with methanol:water (2:1) to a final sample ratio of 100 mg FW mL^−1^. UPLC-MS/MS analysis was performed on a Waters Acquity I class UPLC system coupled to a Waters Xevo TQ-XS (Milford, MA, USA), equipped with a UniSpray ion source (US) operated in timed MRM mode, with argon (Gruppo SIAD, Bergamo, Italy) as a collision gas. Separation was performed on a Waters Acquity HSS T3 column (1.8 μm, 100 mm × 2.1 mm) at 40 °C. For gradient elution, water and acetonitrile containing 0.1 *v*/*v*% formic acid were used. Data processing was performed using Waters MassLynx 4.2 and Target-Lynx software (Milford, MA, USA).

SA extraction was performed according to Pál et al. [76]. After separation on a reverse-phase column (Synergi Fusion-RP, 80A, 150 × 4.6 mm, 4μm, Phenomenex, Torrance, CA, USA) SA was quantified fluorometrically (W474 scanning fluorescence detector, Waters, Milford, CT, USA), with excitation at 305 nm and emission at 407 nm for SA.

### 4.5. Pigment Extraction and Analyses

Pigment extraction and chromatographic analyses was performed as described in [57]. Briefly, liquid N_2_-homogenized 200 mg fresh weight leaf tissue was spiked with beta-apo-8′-carotenal (Merck-Sigma, Darmstadt, Germany) as an internal standard at 2.5 or 5 µg 100 mg^−1^. Samples were extracted twice with 1 mL of acetone:methanol 80:20 *v*/*v*% by vortexing for 10 s, followed by shaking in a MiniG 1600 instrument (SPEX Sam-plePrep.; Metuchen, NJ, USA). After centrifugation at 14,000× *g* (at 4 °C for 10 min), supernatants were collected, pooled, and filtered through 0.22 µm PTFE syringe filters and analyzed immediately. For LC-PDA-MS analysis, a Waters Acquity I-class UPLC coupled to a Xevo TQ-XS mass-spectrometry system and a Thermo Accucore C30 2.6 µm, 4.6 × 150 mm column was used. Eluent system A was methanol:water:tert-butyl methyl ether (TBME) 70:30:30 *v*/*v*%, while eluent B was methanol:TBME 50:50 *v*/*v*%. Solvents used were all at least HPLC grade and were purchased from VWR International (Radnor, Pennsylvania, United States). Absorbance was recorded at 250–700 nm with 1.2 nm resolution and 20 Hz with a PDA detector.

### 4.6. Proline Measurement

The proline content was measured according to the method of Bates et al. [79]. Briefly, the samples were homogenized with distilled water. The extract was centrifuged at 10,000× *g* for 10 min at 4 °C. The supernatant was mixed in a 1:1:1 ratio with ninhydrin acid and glacial acetic acid. The mixture was incubated at 100 °C for 1 h. The reaction was arrested in an ice bath, the chromophore was extracted with toluene, and its absorbance was determined at 520 nm using a UV-visible spectrophotometer (160A, Shimadzu, Kyoto, Japan).

### 4.7. Gene Expression Analysis

For gene expression studies, the second, fully developed leaves and roots of 14-day-old wheat plants were taken and immediately stored in liquid nitrogen. Total RNA extraction and cDNA synthesis were performed as described in Tajti et al. [80]. Briefly, total RNA was extracted from samples using TRI reagent. The samples were treated with DNase I and cleaned with a Direct-Zol RNA MiniPrep Kit (Zymo Research, Irvine, CA, USA) according to the manufacturer’s instructions. For RT-qPCR measurements, a BioRad CFX96 Touch Real-Time Detection System was used with 1 µL 4-fold diluted cDNA, 200 nM forward and reverse primers, 2.5 μL PCRBIO Mastermix (PCR Biosystem, London, UK) and 2.5 μL molecular grade water. Relative transcript levels were determined with the 2^−ΔΔCt^ method [81], with *Ta2291* as internal control gene [82]. Primer sequences are available in Table S1 [64,82,83,84].

### 4.8. Statistical Analysis

Data are presented for the most representative repetition of the four independent biological experiments. The results are the means of 30 replicates for biomass parameters, six replicates for chlorophyll *a* fluorescence induction measurement, and at least three replicates for chromatographic and spectrophotometric determinations. All reactions for gene expression analyses were performed in triplicate using 3 biological and 3 technical repetitions. The data were statistically evaluated using the standard deviation in Microsoft Excel (STDEV.S function) with *n* ≥ 3. Different letters indicate statistically significant differences (*p* < 0.05) between multiple groups (one-way ANOVA with Duncan post hoc test was performed using SPSS 16.0).

## 5. Conclusions

Based on the present findings, light composition under the same light intensity has no pronounced effect on PA content. However, the nearly identical PA levels were accompanied by completely opposite effects of B and R lights on the expression levels of PA metabolism-related genes, with a significant inhibiting effect under the B light, which was only partially compensated under P light conditions due to the presence of a higher proportion of the red component. Although a positive effect of PA treatment was observed under B, R, and P light conditions, as evidenced by leaf biomass parameters and photosynthetic performance, these beneficial changes were correlated only with the increase in endogenous PUT content, especially under B light. Here, PA treatments increased the leaf PUT and SPD contents, as well as the expression levels of genes involved in PA metabolism, confirming that the intensive uptake and metabolism of PAs were responsible for the roborative effects. Despite the fact that initial differences in the expression pattern of PA metabolism-related genes were observed between the three spectral regimes, the effect of PA treatments was opposite under B and R conditions, with a partially intermediate influence under the P light condition. Our results also demonstrated that the effects of exogenous PAs on PA levels were nearly identical under R and P treatments in the leaves. In addition, especially in SPM-treated plants, the leaf transcript levels of *ADC*, *SPDS*, *PAO*, and *pxPAO* genes showed similar changes under R and P light conditions. In the roots, no effect of light spectral composition was observed on the PA treatments regarding PA content, and no similar tendencies in gene expression levels were observed after PA treatments between the three spectral compositions.

**Figure 9:**
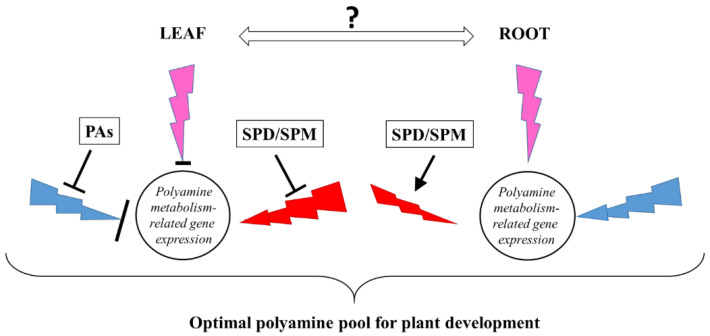
Schematic figure on the effect of polyamines (PAs) (SPD: spermidine; SPM: spermine) treatments in the leaves and roots of plants grown under three light regimes (blue lightning arrows indicate blue light, red lightning arrows indicate red + far-red light, and the pink ones the combination of blue and red + far-red, called pink light) compared to white light conditions without any polyamine treatment.

Taken together, light quality optimizes plant growth under given conditions and may influence plant growth via the adaptive adjustment of PA metabolism at the gene expression level, which results in a similar spectrum-independent PA pattern (Figure 9.). Nevertheless, after PA treatments, the excess of PAs that disturbs the fine tuning of PA metabolism induces different strategies under B, R, or P light conditions for maintaining the optimal PA pool for plant growth and development.

## Figures and Tables

**Table 1:** Color-map comparison of the pigment contents (*trans*-violaxanthin, *trans*-neoxanthin, *trans*-lutein, chlorophyll a, chlorophyll b, *trans*-β-carotene, and 9-*cis*-β-carotene) of leaves of plants grown under different light regimes (white light: W, blue light: B, red + far-red: R and the combination of blue and red + far-red, pink: P) treated with or without polyamines (control: C, putrescine: PUT, spermidine: SPD and spermine: SPM). Values are means ± SD of 3 measurements per treatment. Different letters indicate statistically significant differences at *p* < 0.05 level, using Duncan’s post hoc test.

μg g ^−1^ FW		*Trans*-Violaxanthin	*Trans*-Neoxanthin	*Trans*-Lutein	Chlorophyll *a*	Chlorophyll *b*	*Trans*-β-Carotene	9-*Cis*-β-Carotene
**C**	**W**	57.7 ± 0.4 a	52.4 ± 0.5 g	189 ± 1 d	639. 7 ± 3 bc	280.7 ± 1.2 a	59.3 ± 0.7 c	6.4 ± 0.1 ab
**C**	**B**	77.8 ± 1.8	48.5 ± 1.7 ef	193 ± 3.6 d	714. 3 ± 7.5 e	309. 7 ± 2.5 fg	72.2 ± 1.6 e	8.8 ± 0.1 e
	**R**	54.9 ± 0.8 a	33 ± 0.8 a	157. 7 ± 1.5 a	622 ± 11.1 ab	288. 3 ± 5.5 ab	51.8 ± 0.6 a	6.7 ± 0.1 bc
	**P**	67.7 ± 1.4 d	39.3 ± 0.5 b	178. 7 ± 2.1 c	691 ± 8.7 d	304 ± 2.6 ef	64.2 ± 1.2 d	8.3 ± 0.1 d
**PUT**	**B**	74.5 ± 1.8 e	43.8 ± 1.7 cd	190 ± 4.6 d	746. 3 ± 4 fg	323 ± 1 h	69.4 ± 2.8 de	8.8 ± 0.2 e
	**R**	57.5 ± 1.9 a	38.8 ± 1.3 b	165. 7 ± 5 ab	605 ± 12.5 a	282 ± 5.6 a	51.4 ± 1 a	6.2 ± 0.1 a
	**P**	75.3 ± 0.6 e	45.9 ± 0.8 de	192 ± 1.7 d	725 ± 7 e	316. 3 ± 3 gh	70.4 ± 1.7 de	8.4 ± 0.2 d
**SPD**	**B**	86.8 ± 2 f	52.5 ± 1.4 g	204. 7 ± 4.6 ef	772 ± 15.9 h	331. 7 ± 6.7 i	76.4 ± 0.4 f	9.4 ± 0.1 f
	**R**	62.7 ± 3.3 b	41.5 ± 2.5 bc	176. 3 ± 7 c	651. 3 ± 11 c	294. 7 ± 4.5 bc	56.9 ± 2.7 bc	6.8 ± 0.7 c
	**P**	74.3 ± 0.6 e	44.9 ± 1 cd	190. 7 ± 1.5 d	731. 3 ± 4 ef	318 ± 2 h	70.6 ± 2.4 de	8.3 ± 0.1 d
**SPM**	**B**	84.2 ± 2.5 f	51.5 ± 1.7 fg	209.3 ± 4.9 f	790. 3 ± 12.9 i	346. 7 ± 4.7 j	75.8 ± 1.1 f	9.3 ± 0.2 f
	**R**	62 ± 0.9 b	39.9 ± 0.8 b	171. 3 ± 2.5 bc	656 ± 11.1 c	298. 3 ± 4.5 cd	54.4 ± 0.9 ab	6.6 ± 0.04 bc
	**P**	74 ± 6.5 e	44.1 ± 5.5 cd	204 ± 12 de	755. 7 ± 20.5 gh	330. 7 ± 7.5 i	68.1 ± 4 d	8.6 ± 0.4 de

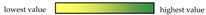

**Table 2:** Color-map comparison of biomass parameters of plants treated with or without polyamines (control: C, putrescine: PUT, spermidine: SPD and spermine: SPM) under different light regimes (white light: W, blue light: B, red + far-red: R and the combination of blue and red + far-red, pink: P). Values are means ± SD (*n* = 30). Different letters indicate statistically significant differences at *p* < 0.05 level, using Duncan’s post hoc test.

		Shoot Length (cm)	Root Length (cm)	Shoot Weight (g ^−1^ plant)	Root Weight (g ^−1^ plant)
**C**	**W**	27.04 ± 1.45 h	18.84 ± 1.93 e	0.31 ± 0.02 a	0.15 ± 0.02 a
**C**	**B**	19.4 ± 0.96 a	16.56 ± 1.44 bc	0.34 ± 0.02 a	0.23 ± 0.02 bc
	**R**	25.8 ± 2.18 g	17.88 ± 4 de	0.38 ± 0.06 abc	0.22 ± 0.04 abc
	**P**	21.56 ± 1.33 c	15.88 ± 2.11 ab	0.32 ± 0.02 a	0.21 ± 0.03 abc
**PUT**	**B**	20.56 ± 1.36 b	17.84 ± 1.62 dc	0.35 ± 0.08 ab	0.22 ± 0.02 abc
	**R**	25.84 ± 1.37 g	18.24 ± 2.22 de	0.45 ± 0.04 d	0.31 ± 0.03 c
	**P**	22.04 ± 1 cd	16.16 ± 2.37 ab	0.35 ± 0.04 a	0.28 ± 0.04 cd
**SPD**	**B**	20.52 ± 1.85 b	14.96 ± 3.12 a	0.33 ± 0.06 a	0.19 ± 0.04 ab
	**R**	24.52 ± 1.69 f	15.76 ± 1.83 ab	0.44 ± 0.03 cd	0.28 ± 0.03 cd
	**P**	22.32 ± 1.63 d	17.6 ± 2.5 cd	0.37 ± 0.0 abc	0.23 ± 0.04 abc
**SPM**	**B**	21.43 ± 1.14 c	15.53 ± 2.26 ab	0.38 ± 0.03 abc	0.2 ± 0.02 ab
	**R**	25.27 ± 1.68 g	14.93 ± 2.36 a	0.47 ± 0.04 d	0.22 ± 0.01 abc
	**P**	23.13 ± 1 e	15.47 ± 1.5 ab	0.379 ± 0.02 abc	0.16 ± 0.01 ab

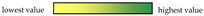

**Table 3:** Characteristics of light regimens.

Treatments	Intensity PAR (µmol)	Blue µW/cm^2^ (400–500 nm)	Green µW/cm^2^ (500–600 nm)	Red µW/cm^2^ (600–700 nm)	Far-red µW/cm^2^ (700–800 nm)	Blue%	Green%	Red%	Far-red%	Blue/Red	Red/Far-red
**White (W)**	250	1120	1840	2240	130	21	34.52	42.03	2.44	0.5	17.23
**Blue (B)**	250	5060	50	1010	10	82.55	0.82	16.48	0.16	5	101
**Red + Far-red (R)**	250	720	680	3420	340	13.95	13.18	66.28	6.59	0.21	10.06
**Pink (P)**	250	2720	20	2690	20	49.91	0.37	49.36	0.37	1.01	134.5

## Data Availability

All data are provided herein.

## Supplementary Materials

The supporting information can be downloaded at: https://www.mdpi.com/article/10.3390/ijms23158394/s1.
