# Supplementary material for: Light Spectral Composition Modifies Polyamine Metabolism in Young Wheat Plants
Source: Int J Mol Sci. 2022 Jul 29;23(15):8394. doi: 10.3390/ijms23158394 (PMC9369354; doi:10.3390/ijms23158394)
Supplement: Supplementary file 1 [file ijms-23-08394-s001.zip › ijms-1792453-supplementary.pdf]

Table S1. Primer sequences.

Ta2291 *ADP-ribosylation factor*	Forward	GCTCTCCAACAACATTGCCAAC	165 bp	[82]
	Reverse	GCTTCTGCCTGTCACATACGC		
TaADC	Forward	AGGAGGAGGAGCTCGACATT	137 bp	[83]
	Reverse	GCCGAACTTGCCCTTCTC		
TaPAO	Forward	CCAGCCTCCAGCTCCGCAAC	137 bp	[84]
	Reverse	GCCCAGCTCCTCCACCTCGTC		
TapxPAO	Forward	GCTCATAAATCAGCCCAATTCCA	113 bp	[84]
	Reverse	TTCGCCATTTGTTGAGCTCT		
TaPUT1	Forward	GGTCTTCTCCCTCTTGCCTT	156 bp	XM_044548016.1
	Reverse	GTGCTGATCGAGTCCCAGTA		
TaPUT2	Forward	TTCATCGCCTTCATCAAGCTG	124 bp	XM_044523314.1
	Reverse	TCACCACGACGATCAGGATAG		
TaSPDS	Forward	AGGTATTCAAGGGTGGCGTG	125 bp	[64]
	Reverse	TGGGTTCACAGGAGTCAGGA		
